# Fatty acids from diet and microbiota regulate energy metabolism

**DOI:** 10.12688/f1000research.6078.1

**Published:** 2015-09-09

**Authors:** Joe Alcock, Henry C. Lin

**Affiliations:** 1 Department of Emergency Medicine, University of New Mexico, Albuquerque, New Mexico, 87131-0001, USA; 2 Division of Gastroenterology, New Mexico VA Health Care System, Albuquerque, New Mexico, 87108, USA; 3 Department of Internal Medicine, University of New Mexico, Albuquerque, New Mexico, 87131-0001, USA

**Keywords:** microbiota, metabolism, diet

## Abstract

A high-fat diet and elevated levels of free fatty acids are known risk factors for metabolic syndrome, insulin resistance, and visceral obesity. Although these disease associations are well established, it is unclear how different dietary fats change the risk of insulin resistance and metabolic syndrome. Here, we review emerging evidence that insulin resistance and fat storage are linked to changes in the gut microbiota. The gut microbiota and intestinal barrier function, in turn, are highly influenced by the composition of fat in the diet. We review findings that certain fats (for example, long-chain saturated fatty acids) are associated with dysbiosis, impairment of intestinal barrier function, and metabolic endotoxemia. In contrast, other fatty acids, including short-chain and certain unsaturated fatty acids, protect against dysbiosis and impairment of barrier function caused by other dietary fats. These fats may promote insulin sensitivity by inhibiting metabolic endotoxemia and dysbiosis-driven inflammation. During dysbiosis, the modulation of metabolism by diet and microbiota may represent an adaptive process that compensates for the increased fuel demands of an activated immune system.

## Introduction

It has long been appreciated that the Western diet—high in simple carbohydrates, processed meat, and fat—is associated with adverse health outcomes, including obesity, metabolic syndrome, and type 2 diabetes ^1, 2^. In particular, consumption of saturated fatty acids and industrially produced trans fatty acids is linked with metabolic syndrome and obesity ^3^. However, controversy continues to surround the relative importance of fat in the diet overall and which fats are healthier or more harmful ^4^. Saturated fat in diet has received much attention for its ability to induce chronic low-grade inflammation, widely recognized as a key link to the pathologies of obesity, type 2 diabetes, and cardiovascular disease ^5^. Dietary fat drives chronic low-grade inflammation by expanding white adipose tissue (WAT), promoting macrophage recruitment to WAT, and generating adipose inflammation (reviewed in 6). Increased release of fatty acids from expanded WAT results in decreased muscle cell surface expression of the glucose transport protein GLUT4, reducing insulin-stimulated glucose uptake and inhibiting glycogen synthesis ^7^. Impaired glucose uptake by GLUT4 is a key feature of insulin resistance (IR), which is a precursor to the development of type 2 diabetes.

Impairment of insulin action and inflammation from dietary fat have been described as resulting from the body’s limited capacity to store energy as fat ^8^. In this view, dietary energy intake in excess of adipose storage capacity causes ectopic fat deposition in non-adipose tissues. Obesity and ectopic fat, in turn, are associated with muscle and liver accumulation of diacylglycerol (DAG) ^9^ and ceramide, a sphingolipid derived from saturated fatty acids such as palmitate ^10^. Toxic lipid molecules, generated through *de novo* synthesis from dietary fat, have pleiotropic effects on metabolism (reviewed in 11). DAG and ceramide have been shown to impair mitochondrial function, inhibit insulin signaling by acting on peroxisome proliferator-activated receptors (PPARs) and protein kinases, and cause inflammation via the nuclear transcription factor nuclear factor **-**kappa **-**B (NF-κB) ^9, 10, 12^.

Despite equal energy content, dietary fatty acids that differ in structure can have opposite effects on inflammation and IR. The divergent fatty acid effects on metabolism cast doubt on a simplistic view of IR as a problem of limited adipose storage capacity. For example, saturated fatty acids but not polyunsaturated fatty acids (PUFAs) caused IR in Sprague-Dawley rats, although both dietary fats resulted in increased plasma-free fatty acids ^13^. Similarly, incubation with saturated fatty acids palmitate and stearate caused IR in human skeletal muscle, whereas unsaturated oleate had opposing effects on insulin action ^14^. In two human trials, substituting dietary saturated fat with polyunsaturated fat or monounsaturated fat improved insulin sensitivity and reduced visceral adiposity ^15, 16^. Certain dietary fats reduce adipose inflammation and IR, even in the overfed state ^17, 18^.

To understand why fats often have opposing metabolic effects, we note that the gut microbiota, the collection of microorganisms that inhabit our bodies and outnumber human cells by an order of magnitude, is sensitive to dietary composition and is linked to changes in metabolism and obesity ^19, 20^. The composition of the diet and gut microbiota interact to modify the risk of many chronic inflammatory diseases, including obesity, diabetes, and inflammatory bowel disease ^21^. The metabolic responses to various fats might be best understood in light of dietary fat’s ability to drive changes in the makeup and function of the intestinal microbiota.

## The relationship of dietary fat, microbiome, and insulin resistance

Recent studies have highlighted the central role of the gut microbiota in generating inflammation and regulating obesity and metabolism ^19^. The microbiota consists of the collection of microbes living in and on our bodies, numbering as many as 100 trillion that reside mostly in the lower intestine ^22^. Advances in sequencing technology and metagenomics have vastly increased the ability to identify intestinal microbes associated with obesity ^23^ as well as mechanisms implicating microbiota in weight gain, such as increased energy harvest ^24^. Compared with germ-free animals, conventionally raised mice have 60% more body fat even as the food intake was less ^25^. This finding was explained by the suppression by gut microbes of the expression of a host intestinal protein known as fasting-induced adipocyte factor (FIAF). Because FIAF is an inhibitor of lipoprotein lipase (LPL), in the presence of gut microbes, less FIAF means reduced inhibition of LPL, resulting in more LPL, the enzyme responsible for importing and storing triglycerides. In a germ-free animal, greater FIAF increased the expression of genes responsible for fatty acid oxidation via stimulation of PPAR-γ coactivator and AMP-activated protein kinase ^26^. Experiments that transferred microbes from obese and lean donors to germ-free mice support a causal role for microbiota in regulating fat mass and metabolism ^27^. In a recent study, fecal microbiota from identical twins discordant for obesity were inoculated into germ-free mice, resulting in the transfer of the obese or lean phenotype of their donors ^28^. Interestingly, when the resulting obese and lean mice were co-housed, the microbiota in lean mice appeared to have a selective advantage, transforming the microbiota of co-housed obese mice and causing weight loss. However, when obese mice were fed a high-fat diet, they could not be “rescued” by co-housing them with their lean counterparts ^28^. In this example and others, an interaction of high-fat diet and specific microbiome appears necessary to cause systemic inflammation ^28, 29^.

## High-fat diet is linked to inflammation and insulin resistance

A diet high in fat is sufficient to induce obesity and IR in many animal models ^4, 30– 33^ and is associated with changes in gut microbiota and intestinal permeability. Two markers of inflammation, tumor necrosis factor-alpha and NF-κB activation, were induced in C57BL/6 mice fed a high-fat diet ^34^. The essential role of the gut microbiota in this response was demonstrated by the absence of this effect in germ-free mice fed the same high-fat diet ^34^. Because inflammatory markers increased before diet-induced obesity, inflammation that follows a high-fat diet may have a causal role in obesity ^34^.

Highlighting the potent effects of dietary fat, a single high-fat meal was sufficient to induce pro-inflammatory signaling and IR ^35, 36^. IR and inflammation following a high-fat meal resulted from increased intestinal permeability to endotoxin ^35, 36^. In addition, because lipid A, the insoluble fraction of the endotoxin lipopolysaccharide, could be carried into the lymphatic system by chylomicrons, a high-fat meal could promote postprandial entry of endotoxin into the circulation even in the absence of increased intestinal permeability. In male C57BL6/J mice, high-fat feeding resulted in weight gain and a two- to three-fold increase in circulating endotoxin, a condition termed metabolic endotoxemia ^4^. Weight gain and IR were equivalent in the group fed a high-fat diet and mice receiving a subcutaneous infusion of endotoxin ^4^. From these results, it was proposed that the Western diet, high in fat and low in fiber, causes a dysbiosis that results in the translocation of gut-derived bacterial endotoxin ^37^. Supporting the role of gut microbiota in this process, IR and weight gain were blocked with antibiotic pre-treatment ^38^. IR by this mechanism involves endotoxin detection by the Toll-like receptor TLR4 and downstream pro-inflammatory signaling ^39– 41^. Recently, Everard *et al.* showed that the metabolic effects of high-fat diet require MyD88 (myeloid differentiation primary response gene 88), a central adaptor molecule for many TLRs with a key role in regulating inflammation and metabolism ^41^. Mice with the MyD88 deletion were protected against high fat-induced metabolic endotoxemia and had increased regulatory T cells, findings that were linked with decreased IR and inflammation ^41^. Additionally, MyD88 deletion altered the composition of the gut microbiota; transfer of those microbes into germ-free mice protected the recipient mice from high fat-induced IR. These results suggest that bi-directional control involving microbiota and the MyD88 pathway regulates metabolism and inflammation.

## Saturated fats are linked to dysbiosis and metabolic endotoxemia

**Figure 1.**
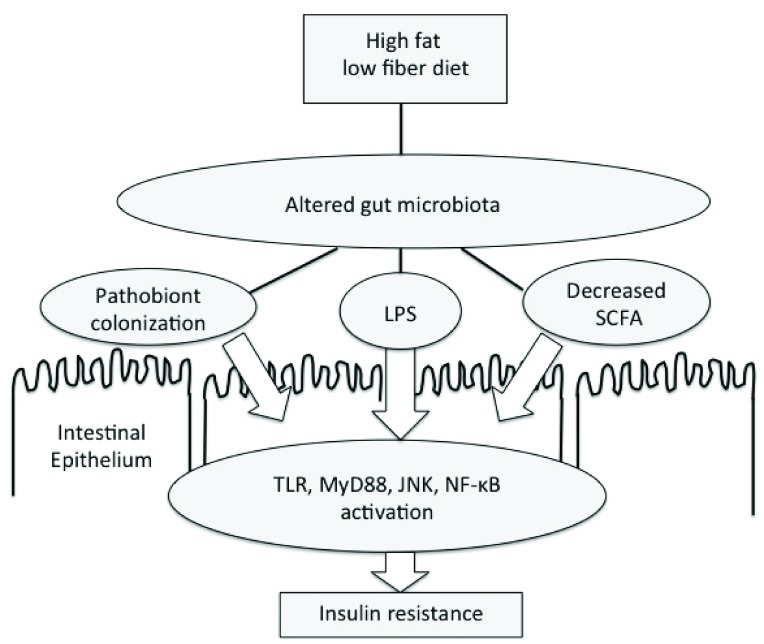
Diet-induced metabolic endotoxemia. Gut microbiota play a central role in the metabolic endotoxemia model of obesity and insulin resistance. Diets high in fat and low in fiber alter the function and composition of the gut microbiota. These changes can increase systemic lipopolysaccharide (LPS) exposure, thereby contributing to low-grade inflammation and impairing insulin-stimulated glucose uptake by muscle. JNK, Jun N-terminal kinase; MyD88, myeloid differentiation primary response gene 88; NF-κB, nuclear factor-kappa-B; SCFA, short-chain fatty acid; TLR, Toll-like receptor.

In this and following sections, we review how specific dietary fats alter the microbiome and change insulin sensitivity. Saturated fatty acids have been shown to have direct stimulatory effects on TLR expression ^42^ and Jun N-terminal kinase (JNK) activity ^43^ promoting IR via mechanisms independent of the gut microbiota. However, these direct effects may be less consequential than the influence of the gut microbiota on host metabolism, as underscored by the finding that germ-free animals are protected from high-fat diet-induced obesity and IR ^25, 34^. Saturated fatty acids have been shown to cause dysbiosis and intestinal inflammation in interleukin-10 ^−/−^ mice by encouraging overgrowth of a bile-tolerant Gram-negative bacteria, *Bilophila wadsworthia* ^44^. In another study of C57BL/6J mice, a diet high in saturated fat caused increased growth of three types of sulfidogenic bacteria, primarily in colonic mucosa ^45^; these bacteria produce hydrogen sulfide gas as a metabolic by-product which can damage the intestinal barrier and cause endotoxemia. Feeding C57BL/6 mice a diet high in saturated fat decreased expression of tight junction proteins, causing increased intestinal permeability, endotoxemia ^46^, and elevated lipopolysaccharide-binding protein ^47^. In addition to higher fecal and plasma endotoxin levels, mice fed a diet high in saturated fat had fewer *Bifidobacteria* and increased Enterobacteriaceae in fecal culture ^46^. Laugerette *et al.* showed an increased intestinal *Escherichia coli* population along with elevated plasma and adipose inflammation in animals fed saturated fat (palm oil) compared with unsaturated fats ^47^. Taken together, these results support the hypothesis that certain diets high in saturated fatty acids may modify the structure and function of the gut microbiota, causing inflammation and IR in animal models ( Figure 1).

## Omega-6 polyunsaturated fatty acids can cause dysbiosis and inflammation

Ghosh *et al.* ^48, 49^ showed that C57BL/6 mice fed a diet rich in omega-6 (n-6) PUFAs (corn oil) resulted in bacterial overgrowth and dysbiosis. The high n-6 PUFA diet, alone among the high-fat diets studied, was also associated with bacterial invasion of the intestinal epithelial cell layer ^48^. Corn oil supplementation caused decreased spontaneous locomotor activity, hyperinsulinemia, and IR in female C57BL/6 mice ^50^. This animal study provides an interesting insight into the “couch potato” sedentary state in humans, suggesting that diet and microbiota can influence voluntary physical activity.

Dietary n-6 PUFAs were linked with changes in the composition of the gut microbiota in C57BL/6 mice ^49^. These changes included increased abundance of Enterobacteriaceae and segmented filamentous bacteria, bacterial groups associated with inflammation ^49^. N-6 PUFA feeding to C57BL/6 mice was shown to increase the numbers of intestinal Proteobacteria ^51^ and change gut microbiota composition along with weight gain and fatty infiltration of the liver ^52^. Huang *et al.* also reported an increase in intestinal Proteobacteria after n-6 PUFA feeding in C57BL/6 mice and greater macrophage infiltration of adipose than observed with saturated fat diets ^53^. Excess dietary N-6 PUFAs caused higher adipose expression of resistin, a hormone linked with inflammation and IR, than was observed after consumption of saturated fat ^53^.

## Omega-3 polyunsaturated fatty acids protect against dysbiosis and promote insulin sensitivity

Ghosh *et al.* demonstrated that altered gut microbiota caused by n-6 PUFAs in 2-year-old C57BL/6 mice was prevented when omega-3 (n-3) PUFAs (fish oil rich in DHA and EPA) were added to the diet ^48^, suggesting that n-3 PUFAs can protect against dysbiosis. N-3 EPA and DHA reversed bacterial overgrowth and reduced fatty diet-induced inflammation by recruiting regulatory T cells to the small intestine ^48^. However, Mujico *et al.* showed no similar protection from n-3 PUFA supplementation from dysbiosis caused by saturated fatty acids ^18^. Another recent study showed that mice fed fish oil had decreased abundance of *Helicobacter* and *Pseudomonas* and Firmicutes, organisms associated with ulcers, infection, and weight gain, respectively ^54^. One mechanism that may account for dietary n-3 PUFA’s reduction of *Helicobacter* and *Pseudomonas* is that those organisms are sensitive to the direct bactericidal effects of EPA and DHA ^55, 56^. Bacterial killing by n-3 PUFAs and other fatty acids is likely important to the overall composition of the microbiota and the function of the intestinal barrier ^57, 58^.

Dietary fish oil strengthened intestinal barrier function and reduced plasma endotoxin levels in swine ^17^. Fish oil has also been linked with reduced TLR activation and MyD88 signaling in swine ^59^. In addition to having beneficial effects on metabolic endotoxemia, n-3 PUFAs were shown to stimulate the G-protein coupled fatty acid receptor GPR120, promoting insulin sensitivity by increasing cell surface expression of GLUT4 ^60, 61^. N-3 PUFAs have additional anti-diabetic effects by activating GPR 40, causing increased insulin secretion from pancreatic β cells.

## Monounsaturated fatty acids antagonize dysbiosis and promote insulin sensitivity

Mujico *et al.* reported that oleic acid (a monounsaturated fatty acid) prevented high-fat diet dysbiosis in female ICR mice and increased the abundance of intestinal *Bifidobacteria*, a group associated with improved intestinal barrier function ^18^. Oleic acid supplementation prevented weight gain and restored the proportion of microbial phyla altered by a high-fat diet ^18^. Hidalgo *et al.* showed that butter produced changes in murine gut microbiota similar to those found in obese humans but that olive oil prevented those changes ^62^. Interestingly, virgin olive oil had different effects on the microbiota compared with refined olive oil, suggesting that the non-lipid phenolic components of olive oil may account for some of its benefits ^62^. Dietary supplementation with monounsaturated oleic acid in young adults improved insulin sensitivity, an effect not seen with saturated fat ^63^. Downstream effects of microbiota may be responsible in part for improved insulin sensitivity and reduced type 2 diabetes observed with diets rich in olive oil and other monounsaturated fats ^64, 65^.

## Short-chain fatty acids and prebiotics promote insulin sensitivity via fatty acid receptors

Short-chain fatty acids (SCFAs) are fully saturated but have fewer carbon atoms than long-chain saturated fatty acids, such as palmitate. SCFAs often have anti-inflammatory signaling properties (reviewed in 57). For instance, SCFAs such as butyrate tend to reduce inflammation by activating the SCFA receptor GPR43 ^66^. GPR43 activation increased energy expenditure and decreased adipose tissue insulin sensitivity while increasing insulin sensitivity in muscle and liver in C57BL/6 mice ^66^. GPR43-deficient mice were obese on normal diet, whereas mice overexpressing GPR43 remained lean on a high-fat diet ^66^. SCFAs are also a by-product of microbial fermentation of indigestible carbohydrates that are termed prebiotics when given therapeutically to alter the microbiota. Prebiotic treatment increased butyrate production in Wistar rats and was associated with increased Bacteroidetes, whereas high-fat diet reduced formation of butyrate and increased liver fat and inflammation ^39^. Butyrate has anti-obesity effects by stimulating the expression of angiopoietin-like protein-4 (ANGPTL4) in human epithelial cells, leading to reduced expression of LPL and increased lipolysis ^67^. Thus, microbes that preferentially generate butyrate through fermentation have a favorable effect on metabolism.

Prebiotic treatment had additional metabolic benefits by increasing the abundance of *Akkermansia muciniphila*, a group of mucin-foraging bacteria that were depleted in obese and type 2 diabetic mice ^68^. *A. muciniphila* separately prevented visceral adipose inflammation, increased anti-inflammatory regulatory T-cell numbers, and improved insulin sensitivity in C57BL/6 mice ^69^. Improvement of glucose tolerance in db/db fiber-fed mice was recently shown to be transmissible with fecal transplantation, even when the recipient mice were never exposed to dietary fiber ^70^. The insulin-sensitizing effects of dietary fiber in donor and recipient mice in that study were attributed to increased *Lactobacillus* and *Bifidobacterium*, decreased *Alistipes*, and changes in amino acid fermentation ^70^. These findings underscore the importance of fiber-fermenting gut bacteria in regulating insulin sensitivity by the action of SCFAs and because of changes in gut microbiota function.

## Discussion

**Figure 2.**
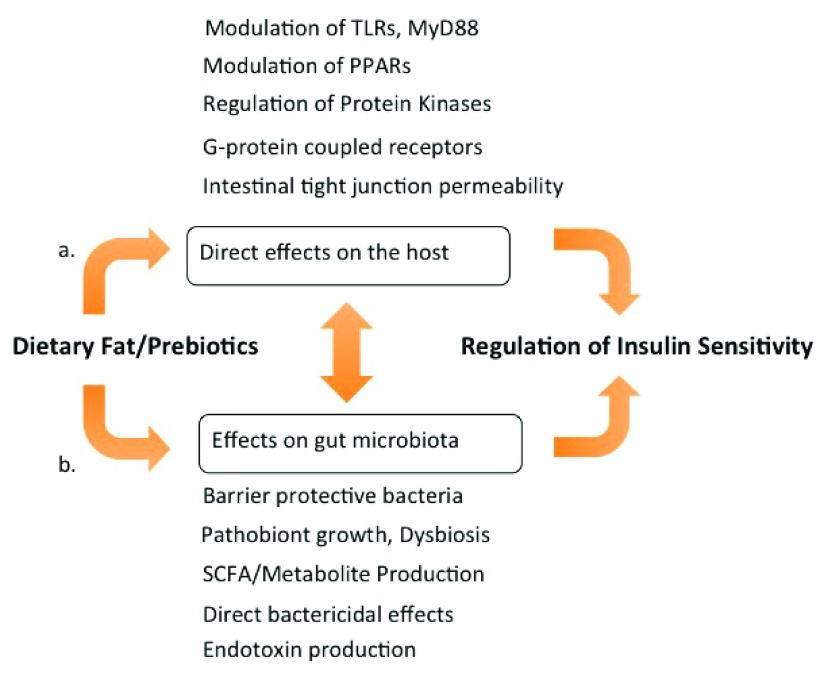
How insulin resistance is regulated by nutrients and microbiota. ( **a**) Dietary fats directly stimulate G protein-coupled receptor (GPR) fatty acid receptors and alter intestinal tight junction protein expression, also affecting the expression and activity of Toll-like receptors (TLRs) and the adaptor protein MyD88 (myeloid differentiation primary response gene 88), modulating nuclear transcription factor activity, and regulating inflammation and metabolism. ( **b**) Dietary fats and prebiotics impact the composition and function of the gut microbiota and affect intestinal permeability. Significant cross-talk occurs between microbiota-derived signals, such as endotoxin and short-chain fatty acid (SCFA), and the pathways described in ( **a**). Cues from both sources, diet and microbiota, are integrated to modulate inflammation and insulin sensitivity. PPAR, peroxisome proliferator-activated receptor.

The studies in this review showing a central role of the microbiota in regulating metabolism and immune function challenge traditional concepts of lipotoxicity as a primary cause of IR and metabolic syndrome ^8^. Explanations of metabolic diseases that center on toxic lipid mediators from overfilled adipose depots are inadequate to explain the widely variable effects of equicaloric fats that have been reviewed here and elsewhere (for example, 71). A more unifying explanation is that the metabolic changes, inflammation, and changes in fat storage leading to obesity are outcomes of dietary fat acting on both the host and the gut microbiota as well as of diet-driven crosstalk between the host and the microbiota ( Figure 2). Protection from obesity and IR in the germ-free state ^25^ and with antibiotic treatment ^38^ provides strong support for this view, which is a departure from the traditional understanding of how metabolic disorders are caused by dietary fat.

It has been suggested that microbiota-induced changes in metabolism can be adaptive for the mammalian host (for instance, by diverting energy to fetal growth during pregnancy) ^72^. The general concordance between insulin sensitivity/resistance and fat-driven changes in the microbiome described in this review ( Table 1) suggests an alternative evolutionary explanation. Specifically, nutrients may serve a signaling function to the immune system in mammals by conveying information about diet-driven changes in the gut microbiota ^57^. This hypothesis makes two predictions: (1) nutrients that lead to dysbiosis may generate pro-inflammatory signaling, and (2) nutrients that prevent dysbiosis may trigger anti-inflammatory signaling. A review of the effects of dietary fats on inflammation and gut microbes tended to be in line with these predictions ^57^. As suggested by the present review, the metabolic effects of dietary fats can often be predicted by their effects on the microbiome, perhaps because metabolism and inflammation share similar regulatory pathways. We further propose that the modulation of metabolism by fats and microbiota may be adaptive in fueling the increased energy needs of immune cells activated by dysbiosis. By blocking glucose uptake, IR reduces energy utilization by tissues dependent on GLUT4 glucose uptake (predominantly skeletal muscle and fat) and diverts energy access to tissues not reliant on insulin-stimulated GLUT4 ^73– 75^. Phagocytes (for example, macrophages) and intestinal epithelial cells do not rely on GLUT4. As a result, glucose energy is expected to be preferentially delivered to activated innate immune cells in the gut during the IR state.

**Table 1.** Dietary fats that cause dysbiosis also cause insulin resistance and inflammation.

Dietary fat	Example (structure) dietary source	Effect on gut microbiota **	Effect on intestinal barrier	Insulin resistance	Inflammation
Saturated fatty acid	Palmitic acid (16:0) * Dairy	Enterobacteriaceae dysbiosis Decreased Bifidobacteria Increased ratio of Firmicutes/ Bacteroidetes [ 18, 44, 53, 68]	Increased endotoxemia [ 17, 35]	Increased [ 68]	Increased [ 30, 35]
N-6 PUFA	Linoleic acid (18:2) Corn oil	Enterobacteriaceae dysbiosis [ 48]	Proteobacteria translocation [ 49]	Increased [ 78]	Increased [ 49, 50]
N-3 PUFA	Docosahexaenoic acid (22:3) Marine fish	Prevented n-6 dysbiosis [ 49]	Prevented Proteobacteria translocation, endotoxemia [ 17, 49]	Reduced [ 60]	Reduced [ 49]
Monounsaturated fatty acid	Oleic acid (18:1) Olive oil	Prevented saturated fat dysbiosis [ 18]	No change [ 17]	Reduced [ 65]	Reduced [ 63]
SCFA	Butyrate (4:0) Dietary fiber	Normalized Firmicutes/ Bacteroidetes [ 68]	Decreased permeability (with prebiotics) [ 39]	Reduced [ 68]	Reduced [ 66]

*Chemical structure (number of carbon atoms:number of double bonds).

**Described in animal models only.

Saturated fats have been reported to cause dysbiosis and have been linked in animal studies with increased Enterobacteriaceae, increased Firmicutes/Bacteroidetes ratio, and decreased Bifidobacteria, among other changes. Dietary omega-6 (n-6) polyunsaturated fatty acid (PUFA) also is reported to cause an Enterobacteriaceae dysbiosis in mice. These microbiota alterations are associated with increased intestinal permeability, insulin resistance, and inflammation. Dysbiosis caused by saturated fat and n-6 PUFA was reversed with supplementation with monounsaturated oleic acid, omega-3 (n-3) PUFA, and short-chain fatty acid (SCFA) precursors (prebiotics). Protection from dysbiosis with oleic acid, n-3 PUFA, and prebiotic supplementation is accompanied by decreased inflammation and increased insulin sensitivity. These patterns are consistent with increased insulin-independent glucose uptake by the activated immune system during dysbiosis and opposite shifts in energy utilization when dysbiosis is absent.

## Ongoing controversies

Despite murine studies suggesting dysbiosis, inflammation, and metabolic disease from n-6 PUFAs, some human studies have shown no harm, and possible benefit, from consuming n-6 fats ^76^. A recent longitudinal cohort study in Finland showed reduced risk of metabolic syndrome with *increased* n-6-to-n-3 PUFA ratio in serum ^77^. Although molecular and animal studies imply a therapeutic benefit of n-3 PUFAs for metabolic syndrome and diabetes, observational studies of n-3 fat and type 2 diabetes have been mixed, indicating a possible reduction of type 2 diabetes risk with fish consumption in Asian populations ^78^ but no benefit from fish consumption in a recent European case control study ^79^. Elevated circulating n-3 fatty acids were recently linked to increased insulin sensitivity in overweight men ^80^ and randomized trials have shown improved parameters related to metabolic syndrome with n-3 PUFA supplementation, including increased adiponectin and improved triglyceride levels in overweight women ^81, 82^. Self-reported diets high in n-3 alpha-linolenic acid, n-6 linoleic acid, and monounsaturated oleic acid have been associated with improved glucose metabolism ^76^. Taken together, these findings indicate possible protection from metabolic syndrome from n-3 PUFAs and support the idea that unsaturated fats are more metabolically healthy than saturated fats. However, a recent study challenged the concept that saturated fats are harmful ^83^. Unlike experiments in which milk fat caused dysbiosis and inflammation in mice ^44^, experiments in humans given a diet high in dairy fat showed no increase in inflammation ^83^. Moreover, improvement in insulin sensitivity and reduced adipose fat occurred more in human subjects assigned a diet low in carbohydrates rather than a diet low in saturated fat ^84^. These results suggest that weight loss and improvements in metabolism can result from diets that prioritize reduction of carbohydrates rather than fats.

One explanation for the disparities between human and animal studies is that people are not mice ^85^ and murine models may be poorly suited to understand human metabolism. Mammalian-microbiota co-evolutionary history is different for humans and other animals ^86^, and a likely consequence is that foods modify the human microbiome differently and have distinct regulatory effects on immunity and metabolism. To date, we cannot define exactly what those species-level differences are. Without that information, it is too early to give a blanket recommendation for or against any class of dietary fat, especially when specific fatty acids in the same class vary in effects and depend also on an individual’s unique microbiota and genetic background. Molecular and human epidemiologic data strongly indicate that some fats are better for metabolic health than others. More human and comparative studies will be needed to determine whether metabolically healthy fats are those that maintain a healthy microbiota.

## Abbreviations

DAG, diacylglycerol; DHA, docosahexaenoic acid; EPA, eicosapentaenoic acid; FIAF, fasting-induced adipose factor; GLUT-4, glucose transporter type 4; GPR, G protein-coupled receptor; IR, insulin resistance; LPL, lipoprotein lipase; MyD88, myeloid differentiation primary response gene 88; n-3, omega-3; n-6, omega-6; NF-κB, nuclear factor-kappa-B; PPAR, peroxisome proliferator-activated receptor; PUFA, polyunsaturated fatty acid; SCFA, short-chain fatty acid; TLR, Toll-like receptor; WAT, white adipose tissue.
