# Supplementary material for: Fatty acids from diet and microbiota regulate energy metabolism
Source: F1000Res. 2015 Sep 9;4(F1000 Faculty Rev):738. [Version 1] doi: 10.12688/f1000research.6078.1 (PMC4797936; doi:10.12688/f1000research.6078.1)
Supplement: Peer review history

# Peer review: Referee response for version 1

**DOI:** 10.5256/f1000research.6507.r10242

**Published:** 2015-09-09

**Authors:** Ted Dinan

**Affiliations:** 1 Psychiatry, University College Cork, Cork, Cork, Ireland

I have read this submission. I believe that I have an appropriate level of expertise to confirm that it is of an acceptable scientific standard.

# Peer review: Referee response for version 1

**DOI:** 10.5256/f1000research.6507.r10241

**Published:** 2015-09-09

**Authors:** Rebecca Pedersen

**Affiliations:** 1 Technical University of Denmark, Lyngby, Denmark

I have read this submission. I believe that I have an appropriate level of expertise to confirm that it is of an acceptable scientific standard.
